# Hospital and Institutional News

**Published:** 1912-12-28

**Authors:** 


### HOSPITAL AND INSTITUTIONAL NEWS.

### THE TRUE KING EDWARD MEMORIAL.

Our position with regard to the latest *volte-face* of the proposals for the London Memorial to King Edward is rather peculiar. As pre-eminently the King most associated with the life and work of the voluntary hospitals, we were more sensitive than any lay journal to the procrastination, the delay, the muddled counsels, and the general want of proper management which, alas, have characterised the proposals from the start. When, evidently as a panic measure, the Committee were frightened by public discontent at their indecision into fixing on the Piccadilly site, we were bound to state " whatever may be the criticisms of this site—and the likelihood of interfering with the present view of the Queen Victoria Memorial is a very obvious one"—that we welcomed the artistic association of the hospitals with other figures in the design. That the Piccadilly site was a bad one even in the eyes of the unfortunate Committee may be taken as proved by their hasty abandonment of it at the first decent chance that offered; and, while being thankful for this, our satisfaction is somewhat chilled by the alternative, which presumably will be accepted by the General Committee, who must by this time be tired of the whole discussion, that—namely, an equestrian statue of King Edward be placed on the ground between the Athenæum and the United Service Clubs. For hospitals can hardly be associated with an equestrian statue. However we do not press this point as the true King Edward Memorial, so far as the hospitals, we had almost said the nation, are concerned, is King Edward's Hospital Fund for London, which will be a living memorial so long as the hospital system which the late King Edward assisted in creating endures. As a sidelight on history we invite the curious reader to compare the wealth of suggestion for the London Memorial that prevailed in October 1910 with the final decision recorded this week. The former we summarised in The Hospital of October 8, 1910. The difference between the original suggestions and the final result will not be lost on the observant critic of society.

### NOISE IN THE WARDS.

To the sensitive stranger, as we hinted inferentially last week, the noise in the wards which signalises Christmas Day, except for that solemn interval which usually is enforced after the patients' Christmas dinner, must seem a very doubtful blessing. The serious cases, of course, for the most part are removed to a separate ward, but it is nevertheless true that patients apparently too ill to care for any interruption, much less the organised din of singing and music, are to be found in the wards visited by the minstrels. It has been a habit of ours for many years to spend the best part of Christmas afternoon in different hospitals, and our experience on this point is perhaps worth having. We have questioned several—the most seriously ill—patients at different times as to how they liked the noise, hinting pretty plainly that we should not be at all surprised to hear that it was the last thing for which they wished. Where the patient had not passed beyond caring for the fact of existence at all the answer was invariably a smile, accompanied by a shake of the head at our suggestion of disapproval. It seemed as if it was much to these patients to know that Christmas was being kept even if they might not take any part in the keeping, not even that of raising the head to view the singers. In the same way people who really prefer the summer and dislike the cold often profess to complain of absence of snow and biting wind in December, and ridicule its stray sunshine and mild temperature as unseasonable and therefore unwelcome. We judge our pleasures for the most part by habit, and not by the joy that they actually give us. And sick patients like the noise in the wards for the same reason as chilly people like a cold winter. Both expect it, and would feel annoyed if their expectations were unfulfilled.

### THE SAXON SNELL PRIZE WINNERS.

Acting upon the advice of the adjudicators and of the consulting referee, the Council of the Royal Sanitary Institute have decided to divide the Henry Saxon Snell Prize of fifty guineas, giving one half the sum to Mr. John Darch, M.R.San.I. (Wandsworth), writing under the motto " Aseptos," and the other half to Mr. H. F. V. Newsome (Manchester) and Mr. John G. Cherry, M.R.San.I. (Manchester), writing jointly under the motto " Magnum Bonum." It will be remembered that the subject given in 1912 for the essay in competition for this prize was: " The Ventilating, Lighting, Heating, and Water Supply Appliances and Fittings for an Operating Room for a General Hospital." Ten essays were sent in. The adjudicators for the competition were Mr. Edwin T. Hall, F.R.I.B.A., Dr. Louis C. Parkes, and Mr. A. Saxon Snell, F.R.I.B.A.; and they had the advantage of criticisms and suggestions made by Sir Frederick Treves, Bart., G.C.V.O., who acted as consulting referee. A bronze medal of the Institute will be awarded to each of the successful competitors. The adjudicators also desire to commend the essays sent in under the mottos: " Science moves but slowly, slowly creeping on from point to point," " Ajax," and " Tout bien ou rein."

### ALEXANDRA DAY AND ITS PROCEEDS.

Attention has been drawn in *Truth* to two serious facts concerning the proceeds of Alexandra Day, which demand public explanation. The first is that, according to the sums published in the newspapers, the net total has diminished since its collection by something like one-half, and the second is that not till last week did one hospital receive its share at all. Those responsible for the collection and administration of the fund have since come forward to explain that the apparently unaccountable dwindling of the sum total was due to exaggerated reports in the lay press, but the delay in its disbursement is not so satisfactorily accounted for. Hospitals have a right to demand an account of the stewardship of the officers of these funds, for it is sometimes too lightly supposed that the needs of charities can be exploited by collectors, and that, provided some sum of money is eventually handed over to them, the charities concerned can be relied on for nothing but thanks. This is not the case. The public are induced to subscribe by the announcement that the collection is on behalf of hospitals. The total raised is flaunted abroad in the press to show the honour due to the collectors, and then a long and unbusinesslike interval is allowed to ensue. We hope that *Truth* will be equally successful in pressing for a complete explanation.

### HOSPITAL SERVICE IN THE CARDIFF SYNAGOGUE.

Although the Hebrew community in Cardiff have always been active and generous in assisting King Edward VII.'s Hospital and Hospital Sunday it was not until this year that a definite Hospital Service was arranged by the synagogue. The first Hospital Service was held last Sunday week in the Synagogue, Cathedral Road. The Lord Mayor of Cardiff, Alderman Morgan Thomas, was present, accompanied by members of the City Council; and representatives of the hospital board of management included the Chairman, Major-General Lee, and Mr. Rea, the secretary. A powerful sermon was preached by the Rev. A. A. Green, minister of the Hampstead Synagogue, London, who based his remarks on Malachi's question: "Have we not all one father? hath not one God created us?" The preacher's appeal was generously responded to, and it is likely that the service will become an annual event, which might well be imitated in other centres.

### MEDICAL WOMEN AT DINNER.

In proposing the toast of "The Royal Free Hospital and London School of Medicine for Women" at the annual dinner of the school last week, Mr. Stanley Boyd, F.R.C.S., who was in the chair, referred to the fact that for the first time for many years the school was free of debt. Thirty-seven new students entered in October last, and the present total of 180 students was the largest number in the school for many years. During the past year, moreover, several valuable scholarships, bursaries, and prizes had been endowed. The Chairman's references to the enthusiastic pioneers, Mrs. Garrett Anderson, the president of the school, and Mrs. Scharlieb, were received with more than usual warmth at a time when the proposed scheme for a South London Hospital for Women is flushed with the possibilities opened up by the gift of £25,000 which was announced in our last issue. This, the eleventh annual dinner, was therefore more deeply an occasion for medical women,—we would much rather use the classical English term, doctresses—to dine *en fête* than ever.

### THE WORCESTER INFIRMARY MEMORIAL.

The City of Worcester "King Edward Memorial Fund," which is now closed, has handed a balance of some £50 to the general infirmary, but the secretary, in answer to an inquiry, explained that there remains about £300 to be paid for the actual building which forms the hospital's extension and memorial. It is not so easy, though perhaps more important, to record the end and completion of hospital memorial schemes as it is to hear news of their inauguration. As with all efforts that spring from a sudden impulse, some schemes have come to a triumphant completion, some have failed like the corn that was sown on the rock, and some are dragging on to an indefinite conclusion. Sir James Gildea, who was commanded by Queen Alexandra to compile a list of completed memorials to the late King, must have found this out in a task that requires infinite energy and patience for adequate fulfilment. The Worcester memorial is fortunate enough to have completion in sight.

### MR. CHAMBERLAIN SEIZES THE MOMENT.

Either Mr. Austen Chamberlain must rank as one of the most successful appealers of recent years, or else his cause, the London School of Tropical Medicine, must be regarded as usurping the public's old-tried and intense affection for the trinity of most popular pleas—children's hospitals, cancer research, and, of late, tuberculosis: perhaps it would be invidious to decide. At all events so prompt and generous has proved the public's response to the recent appeal for £50,000 for the London School of Tropical Medicine that, contemptuous of so cheap a victory, Mr. Chamberlain is now slyly asking for £100,000. A masterpiece needs not only the man but the moment, said Matthew Arnold, and Mr. Chamberlain has certainly proved the man to seize the moment in the present case. Hospital committees who are tremblingly preparing an appeal are commended to a study of his success.

### THE LATEST NOTIFIABLE DISEASE.

The Local Government Board announces that from February 1, 1913, non-pulmonary tuberculosis will be included in the list of compulsorily notifiable diseases. This extension is one which experts have been pressing for some time, and it is merely the logical sequence of the compulsory notification of tuberculosis of the lungs. It is true that the sufferer from joint or intestinal tuberculosis (or from any other of the protean forms of the infection) is less dangerous to his fellow-citizens than he who is expectorating large quantities of tubercle bacilli daily. But from the point of view of obtaining reliable and comprehensive statistics about the incidence of tuberculosis, and of controlling the measures most likely to diminish its incidence, the contemplated extension will undoubtedly be of great service to the public health.

### SANATORIA FOR LONDON: FRESH DECISION.

Only a fortnight ago we commented favourably upon the project for co-operation between the London County Council and the Metropolitan Asylums Board over the sanatorium treatment of insured persons in London. The project, it will be remembered, was for the conversion to sanatorium purposes of a smallpox isolation hospital at Dartford, to be staffed and managed by Asylums Board officers, subject to the supervision of the Council. We are now able to announce that this plan has been rescinded at a special meeting of the Asylums Board held on December 20, in favour of another one. By the new arrangement the Downs School at Sutton is to be allocated to sanatorium purposes, instead of the hospital at Dartford. In some respects the new scheme possesses advantages over the old one, though it has also corresponding drawbacks. One of the latter is that the process of conversion will take longer, because the building is in itself not so well suited to its new purpose. We trust that there will be no penny-wise-pound-foolish economy over these alterations, that nothing but the highest standard of sanatorium construction will be tolerated, and that the superintendent will be a medical man entirely familiar with the treatment and management of consumptive patients.

### TREATMENT BY COMMITTEE.

The South Staffordshire Joint Smallpox Hospital Board has recently converted its institution into a consumption hospital, from which three patients recently departed without permission "from a feeling of homesickness." In answer to inquiries stating that an unfortunate rumour was noised abroad to the effect that the patients received insufficient food, Dr. Lee, the resident medical officer, explained that over one hundred visitors arrived on the first visiting Sunday, and that the memories which their presence excited in the three patients had led them to leave without his sanction. He had made personal inquiries of every remaining patient as to whether they had any complaint to make of the food, and two suggested that they would like eggs and bacon for breakfast. As that was a small item he had readily granted it. Dr. Lee added that there had been an all-round increase in weight, which showed that the diet was suitable. Mr. W. Fletcher, a member of the Wolverhampton Board of Guardians, who had raised the question of the patients' diet at the meeting, said that as they were at the institution only for six weeks their food should be good. At the workhouse infirmary, of which he had always been proud, eggs and bacon were given to similar patients for breakfast, and he suggested that a committee should be appointed to draw up a dietary table for the patients. Dr. Lee's explanation, however, appeared to satisfy him, and the matter then dropped. While sympathising with the tactful manner in which Mr. Fletcher made his remarks, we are glad that his suggestion for the appointment of a committee to draw up a diet table for the patients was not adopted. Such a table must be under the sole control of the resident medical officer, and it is characteristic of a Guardian's point of view in general that the appointment of a committee should be the first expedient to occur to him. Guardians revel in committees, though they must often see little result from them, and, however expedient such committees may be for general business, it would be a bad day for patients and doctors if treatment were transferred to them from the hands of the medical men.

### THE "ALEXANDRA DAY" FUND VERSUS EALING HOSPITAL.

Another aspect of the "Alexandra Day" Fund question is seen in the following letter:—A correspondent writes: "The borough of Ealing was one of the few districts outside London that celebrated 'Wild Rose Day' with any enthusiasm, and considerable dissatisfaction has followed the announcement respecting the distribution of the "Alexandra Day" Fund. Whilst Acton and Putney Hospitals each receive £100 and Tottenham £200, the claims of Ealing's King Edward Memorial Hospital have been overlooked; and it is a keen disappointment to those who laboured to raise the large sum paid over by Ealing to the Fund to find that their own hospital reaps no advantage." Many predict that this will lessen the interest by Ealing residents in "Wild Rose Day" next summer. Where are the accounts and the complete list of awards of this Fund?

### THE CINEMATOGRAPH AND THE ENGLISH SUNDAY.

The cinematograph entertainment is proving the *enfant terrible* of the theatrical, political, social, and institutional world with startling results. It has raised higher dividends than any form of theatrical entertainment on a popular scale of recent years, including even all the variations played on the popular perversion of the Salome dancing theme; it is now rivalling the variety theatre, as the latter in its music-hall days rivalled the legitimate theatre; and finally its present absence of vocal illusion enables it to place on view persons usually excluded by Royal prohibition from trespassing upon the stage, as the topical "From Cross to Manger" films are serving to show. It is also beginning to Continentalise the Victorian English Sunday, for the exclusion of popular entertainments from this day cannot be more than 150 years old. And it is this latter issue which brought the cinematograph into its first relation with hospitals, though the bacteriological films and the still more recent adaptation of the apparatus to entertainments in private premises—which the wards of several London hospitals are trying for the first time during Christmas week—are tending to make the relation less undesirable and more satisfactory in most respects. The national health is also affected by the granting of such seven-day licences to cinematograph theatres as the Brighton Town Council has now decided on, though, provided that the condition can be enforced, their proviso that all employees should receive one day's holiday a week should remove all serious objection to the scheme on this score. We have always maintained that one day's rest in seven was an essential minimum of rest.

### THE MODERN LEPER AT CARDIFF.

The trend of events as regards the progress of the Welsh Memorial to King Edward has offered much interest from the early days when it was proposed that the extension of the then Cardiff Infirmary should be associated with it. There have always been, as no doubt was inevitable in the case of a scheme so gigantic as the Welsh Memorial, many side-issues, a few differences, and withal a steady flow of public money towards the main object. For instance, it is now announced by the executive committee, of which Mr. David Davies, M.P., is president, that the promises and donations amount to over £200,000. As an instance of the problems arising out of the side-issues of the national anti-consumption campaign in Wales, it is interesting to record that the proposal to purchase Grove House, Cardiff, for a consumption hospital has been abandoned. The local resentment excited by this proposal has not figured largely in our columns because observation seemed to show that the hostility of the local property-owners and other interested parties was of the same nature as that equally loudly expressed in Ireland recently in a somewhat parallel case—that is to say, it appeared to have its root in unseemly panic. The fact is that, like most movements, the anti-phthisis campaign, whether in Wales or elsewhere, has another side. We fear that the ignorance which its warnings have no doubt helped to dispel has also been accompanied by an altogether exaggerated fear of the disease's infectivity. A new ignorance, in other words, has taken the place of the old, an ignorance which actually places on a par the establishment of a sanatorium in an urban area with the presence of an infected person in an overcrowded house. No confusion of thought could be greater than this, which has led to consumptives being treated like lepers, not merely by hotel-keepers, but even by public bodies. We wonder which ignorance, the old or the new, Dr. Marcus Paterson, the director of the Welsh campaign, finds the more destructive of real progress?

### THE WAGES OF MEDICINE.

Cries of rapacity and accusations of wealth have been prolific against the medical profession since the Insurance Bill became an Act in July; whether it will succeed in becoming a fact in January is still an open question. We have done our best to puncture this delusion by not merely printing, as The Hospital has done for many years, the estates which medical men leave as soon as probate has been granted, but by drawing attention in a more marked manner to the relative poverty that these reveal when compared with the amounts realised by members of sister professions. It is sometimes argued very truly that the Services, whether school, public health, or Army and Navy, are better indices to the economic status of the rank-and-file of the various professions than any other one factor, because payment in the public service is generally a conservative reflection of the rewards which open competition leads intending candidates to expect, while negativing the possibility of the best and worst incomes in it. What, then, is the lesson to be learnt from the announcement that Inspector-General Sir Herbert Mackay Ellis, R.N., has left unsettled property of the gross value of £15,171, of which £14,926 is net personalty? It is that the head of the Navy Medical Service is expected to have accumulated at the time of his death savings amounting to five or six hundred a year. That being so, less highly paid medical men will be expected, and are found, to think themselves very well off indeed with even half this capital. Would a lawyer be satisfied with this prospect? Would a politician be grateful for such an end to his "career"? Would even an engineer, architect, or moderately successful novelist be ready, like George Borrow, to create and bring up a numerous progeny on these terms? No; the prospect is left to the rapacious and money-loving doctor. Everyone else declines with thanks, for the wages of medicine are like the wages of sin, at least in regard to contract practice.

### THE GROWTH OF HOSPITAL SOCIETIES.

The latest addition to the town and district hospital societies, whose object is the systematic collection of working-men's contributions, is that now provided at Cardiff. A full account of the new organisation appears in another column. But, apart from details, we wish to emphasise the value of example and publicity in the life of such organisations. We understand that one of the main inspirations of the new Cardiff Hospital Society has been the great success achieved by the Leicester and County Hospital Society, by means of which the working-people's contributions were increased, we believe within a decade, from £4,000 to £14,000 per annum. Cambridge again, with its Town and District Workers' Hospital Fund, has shown how county towns in non-manufacturing centres can achieve similar success. The Hospital may take credit for supporting these movements from the first—in the early days when critics were many and supporters few; and by the steady publicity which it has always given to their affairs, may take credit also for spreading the valuable lessons that these movements have taught, and so encouraging new centres to profit from them. Luckily the secretaries of these funds have seen the value of recording each step of their progress, and their reward for the trouble this entails is seen in the growing number of their imitators.

### THE ONLY MEDICAL PEER.

The institutional world as well as the medical profession will have noticed with very genuine apprehension and regret the serious bulletins which have been published for some days concerning the state of Lord Ilkeston's health. Since Lord Lister's death early this year, Lord Ilkeston has been the only representative of the medical profession in the Upper Chamber; and although the bestowal of his peerage was not wholly, nor even mainly, for distinction in medicine, his lordship's record in the profession was of no ordinary kind. The citizens of Birmingham do not need to be reminded that as Walter Foster he was Professor of Medicine there for twenty-five years, as well as physician to the General Hospital, and to two or three special hospitals. In view of the present conflict between the medical profession and the Chancellor of the Exchequer, it is interesting to notice that as long ago as 1883 Dr. Foster, as he then was, wrote upon the "Political Powerlessness of the Medical Profession." We cannot help feeling that had Lord Ilkeston remained in good health and in the confidence of the present Government, his robust common-sense and knowledge of medical practice would have gone far towards solving the difficulties which the Insurance Act as at present administered has created.

### DEFINITIONS OF DRUNKENNESS.

The recent case of Driver Knox, which led to the victorious strike on the North Eastern Railway, brought into view the need for discriminating between being the worse for drink and being drunk; the distinction is important, as coroners and police magistrates well know, and we agree with the City Coroner that it is a pity that medicine cannot, or at all events has not, provided a uniform test. The varying standards which obtain amount in practice to a looseness of definition which allows a prosecutor to stigmatise his learned friend's client as drunk, while the defendant counsel will allege being the worse for drink by way of palliation. "The worse for drink" is really such a beautiful piece of descriptive writing, it does actually sum up a well-known condition so tersely and well, that it is a pity some piece of evidence or symptom cannot be regularly attached to it. The late Samuel Butler used to remind scientific writers, however, that "definitions are like steps cut in a steep slope of ice or shells thrown on to a greasy pavement; they give us foothold and enable us to advance, but when we are at our journey's end we require them no longer." As he quotes Sir William Grove as saying of thought, there is many a thing "so obvious to simple apprehension that to define it would make it more obscure." Let us all, therefore, beware before trying our wit on a definition of drunkenness, though Sir William Grove's admirable common-sense warning fails when opposing parties have an interest in disagreeing over a simple phenomenon of this kind.

### AMBULANCE AND THE TRAFFIC PROBLEM.

It seems amusing at first sight to learn that "in consequence of the large number of street accidents which are daily occurring" in Willesden, in which motor-cars are still held by the public at large to be the primary cause, the Willesden Council have decided to place an additional motor service on the streets. The service in question is, of course, a motor-ambulance service, and, in view of the unsatisfactory condition of London in respect to ambulance services, in many points the proposal is worthy of respectful gratitude. Leaving the hard issue of London's ambulance services on one side for the moment, it may be worth while to dispel the illusion that still delights to throw all the blame for street accidents on motor vehicles. There is really only one condition which may be said basically to safeguard the public from street accidents, and that is that the traffic congesting the streets shall be of the same motive power and relative speed. Horse-drawn vehicles produce their fair average of accidents, in proof of which the various Motor Unions were compelled in self-defence to produce statistics, and did so finally without much trouble; so do motors. Indeed, honours are easy between the equipage and the autopage (to coin a convenient word which etymologically appears to be excrementitious). The true cause of a high ratio of street accidents is a mixed traffic. At present we have the swift motor-car, the slow coach, the insidious bicycle, and the pre-occupied pedestrian: a hotch-potch combining all the ingredients of disaster. The motor has come to stay, for town traction at any rate, and the moral is to level up to its rate of speed and controllability.

### HURRY UP, INSTITUTIONS.

Why does not some newspaper start a holiday competition for the best answer to the question: On which day of the week is it most inconvenient for Christmas Day to fall? Our answer unhesitatingly would be "Wednesday," partly because that is our usual press day, which in consequence had to be shifted to Monday, December 23, but chiefly because it compels us to hold over till next week our accounts of the Christmas festivities in the wards of provincial and London institutions. There is compensation, however, to be found in the fact that, if our readers will only co-operate, the additional time that chance gives to them for describing their Christmas entertainments should enable much fuller and more literary descriptions to appear. We want this year the general public to enter into the spirit of hospital Christmas, and we, therefore ask every kind of institution—the hospital, the Poor-Law infirmary, the sanatorium, the mental hospital, the cottage hospital—to do its best in sending us a full account. There is plenty of time, since in order to appear in our next issue, dated January 4, contributions describing the Christmas festivities must arrive not later than Monday next, December 30. We hope that this year we may present an unrivalled picture of Christmas in the institutional world. Every accepted contribution will be paid for; time is ample, and the chance is a really great one for bringing home to the public the last Christmas which the voluntary hospitals will keep before the dreaded Insurance Act changes their conditions to the core.

### THE IDEAL NEW YEAR PRESENT.

The really magnificent enthusiasm which characterised the Bishop of London's speech at the annual meeting of the League of Mercy, which we published as a special article last week, has evoked a similar response in the hearts of thousands of readers. To meet their demand we have therefore reprinted the speech under the title of "The Poor, our Voluntary Hospitals and Nurses," on antique paper in pamphlet form, and it can be obtained, price threepence, from The Scientific Press, Ltd., 28 and 29 Southampton Street, Strand. In its new attractive form it makes the ideal New Year present, for it not only combines the charm of a New Year card with thoughts of a magnetic personality, but by reproducing so eloquently the atmosphere of a hospital Christmas it carries the lessons of the past's enthusiasm into the opening of an anxious year. It will remain for ever a record of the voluntary hospital spirit, and will brighten the depressed institutional worker whenever he turns again to its pages, as he assuredly will during the ensuing months. For mere eloquence this speech was one of the very best we have heard the Bishop of London deliver, and we have heard him address all sorts of audiences at all sorts of times. We believe that the speech will live as long as men care for charity or eloquence, and our readers will appreciate it all the more in its attractive pamphlet form.

### THE BEST CHRISTMAS CARD OF THE YEAR.

As this is the last Christmas which the voluntary hospitals will keep under the old conditions, for the Insurance Act effect cannot be known, we have a special wish to give to our readers. We cannot take the credit of the phrase in which it is expressed, as that forms the motto on the Christmas card that a public man is sending out to his friends this year, among whom we are fortunately counted. Below a calendar, showing that the succeeding motto is intended to apply to every day in the year, is painted an English bull-dog, squatting on his master's whip, and underneath is written: "When troubles arise, promptly sit on 'em." As a motto for the hospitals in 1912 this could hardly be beaten, and in all sincerity we commend the advice it contains to our readers in the present crisis. Among the welter of inane Christmas conventionalities this motto is a clarion call to the brains and energies of all. That it will wear well we have no doubt.

### PUBLIC VACCINATORS AND SUPERANNUATION ALLOWANCES.

A somewhat novel request has been made to the Southwark Board of Guardians by one of their public vaccinators for the return of the deductions from his fees under the Poor-Law Officers' Superannuation Act, 1896. In a recent decision Mr. Justice Neville held that Guardians were not empowered to grant superannuation allowances in respect of vaccinators' fees. The sum deducted by the Southwark Board of Guardians from the fees amounts to £286 17s. 8d. It has been decided to submit the matter to the Local Government Board, the doctors agreeing to abide by the latter's decision. The Board is to be asked whether the district public vaccinators or the institutional medical officers are entitled to claim (when the time arrives) superannuation allowances under the Act in respect of vaccination fees. Should the answer be in the negative, the Board is requested to state whether there is any objection to the Guardians returning the deductions made from the vaccination fees of the doctors concerned. It is a question quite worth settling.

### THE VALUE OF A MAN.

A Berlin doctor once calculated in the *Revue Internationale Illustrée* the sum necessary to reconstruct a man according to the latest data of science and industry. A pair of arms of good quality cost about £14; the same pair of arms with properly articulated hands £35. A pair of legs are worth £28. The price of a properly constructed nose comes to between £16 and £20. It is possible to deliver a pair of ears with resonators for a trifle of £28 or so, and a dentist would be prepared to furnish an excellent set of teeth for £12. Two artificial eyes of the latest and most up-to-date pattern could hardly cost more than £6. In short, to have a complete human being it would be necessary to disburse some £120. The German physician is not, however, prepared to guarantee that the association of all these divers parts will result in that mysterious abstraction—life!

### THE INVALIDS' FRUIT AND THE CHRISTMAS MARKET.

The fact that grapes are the proverbial gift of anxious relatives to invalids near and dear to them lends interest to the state of the fruit market this Christmas—always a season at which presents of edibles, including fruit, come in particularly large quantities to hospitals. Apart from this, too, the Christmas Festivities Fund has to make purchases, and may have experienced the truth of a statement, said to have been inspired by an "expert" at Covent Graden, that "nothing is dear in fruit this Christmas." Apples, from the same source it appears, are cheap and abundant, though why the buying public continues to neglect the less gaily-coloured but far sweeter English home-grown varieties, with their beautiful rough rinds, small cores, and true tang in the mouth, can only be explained by the fact that the public is a monster with no standard of taste or beauty, the dupe of every rosily-polished Colonial pippin. Oranges are reported to be cheap, but with apples and other fruits may not have proved so to the more prudent institutions, whose Festivities Funds are a well-recognised annual feature. For the early purchases which prudence enjoins proved expensive this year owing to the outbreak of war in the Balkan Peninsula, which caused a rise in prices, among dried fruits at any rate, through the fear lest there might be a scarcity for the Christmas market, though the advent of fresh supplies brought prices down again last week. But dried fruits are generally connected with the idea of the Christmas plum-pudding, which have no reason, so far as the fruit ingredients are concerned, to fear competition with those of yester-year.

### TO OUR READERS.

Contributions are specially invited from any of our readers to these columns. They should deal with topical subjects and news. They must be authenticated for the information of the Editor only. The minimum payment if published is 5s. There is no hard-and-fast rule as to space, but notes of about twenty lines in length are preferred.

